# Regional cerebral blood perfusion changes in chronic stroke survivors as potential brain correlates of the functional outcome following gamified home-based rehabilitation (IntelliRehab)—a pilot study

**DOI:** 10.1186/s12984-022-01072-w

**Published:** 2022-08-24

**Authors:** Younis M. S. Firwana, Mohd Khairul Izamil Zolkefley, Hasnetty Zuria Mohamed Hatta, Christina Rowbin, Che Mohd Nasril Che Mohd Nassir, Muhammad Hafiz Hanafi, Mohd Shafie Abdullah, Bilgin Keserci, Natasha A. Lannin, Muzaimi Mustapha

**Affiliations:** 1 Department of Neurosciences, School of Medical Sciences, Universiti Sains Malaysia, 16150 Kubang Kerian, Kelantan, Malaysia; 2 Rehabilitation Unit, Hospital Universiti Sains Malaysia, Universiti Sains Malaysia, 16150 Kubang Kerian, Kelantan, Malaysia; 3 Department of Radiology, School of Medical Sciences, Universiti Sains Malaysia, 16150 Kubang Kerian, Kelantan, Malaysia; 4 Department of Neuroscience, Central Clinical School, Monash University, Melbourne, Australia; 5 Faculty of Industrial Sciences and Technology, Universiti Malaysia Pahang, Kuantan, Malaysia; 6 Kulliyyah of Islamic Revealed Knowledge and Human Sciences, International Islamic University Malaysia, Kuala Lumpur, Malaysia

**Keywords:** Telerehabilitation, Arterial spin labeling, Cerebral blood flow, Upper extremity, Chronic stroke

## Abstract

**Background:**

Hospital-based stroke rehabilitation for stroke survivors in developing countries may be limited by staffing ratios and length of stay that could hamper recovery potential. Thus, a home-based, gamified rehabilitation system (i.e., IntelliRehab) was tested for its ability to increase cerebral blood flow (CBF), and the secondary impact of changes on the upper limb motor function and functional outcomes.

**Objective:**

To explore the effect of IntelliRehab on CBF in chronic stroke patients and its correlation with the upper limb motor function.

**Methods:**

Two-dimensional pulsed Arterial Spin Labelling (2D-pASL) was used to obtain CBF images of stable, chronic stroke subjects (n = 8) over 3-months intervention period. CBF alterations were mapped, and the detected differences were marked as regions of interest. Motor functions represented by Fugl-Meyer Upper Extremity Assessment (FMA) and Stroke Impact Scale (SIS) were used to assess the primary and secondary outcomes, respectively.

**Results:**

Regional CBF were significantly increased in right inferior temporal gyrus and left superior temporal white matter after 1-month (p = 0.044) and 3-months (p = 0.01) of rehabilitation, respectively. However, regional CBF in left middle fronto-orbital gyrus significantly declined after 1-month of rehabilitation (p = 0.012). Moreover, SIS-Q7 and FMA scores significantly increased after 1-month and 3-months of rehabilitation. There were no significant correlations, however, between CBF changes and upper limb motor function.

**Conclusions:**

Participants demonstrated improved motor functions, supporting the benefit of using IntelliRehab as a tool for home-based rehabilitation. However, within-participant improvements may have limited potential that suggests the need for a timely administration of IntelliRehab to get the maximum capacity of improvement.

## Introduction

Cerebrovascular disease (CVD) such as stroke is the third leading cause of death in the United States, Canada, Europe, and Japan. The American Heart Association and the American Stroke Association estimated that approximately 800,000 new strokes occur each year, resulting in more than 130,000 annual deaths in the U.S. alone [1]. CVD is also the second leading cause of disability-adjusted life-years (DALYs) [2]. In Malaysia, the incidence rate for both hemorrhagic and ischemic stroke has increased annually by 18.7% and 29.5% respectively, making stroke as the third leading cause of mortality [3].

Previous studies had shown that lesion localization influences the functional outcome of stroke patient, hence, different infarct locations will lead to different pattens of brain injury and functional reorganization [4]. Also, the blood supply in the involved regions may be affected by the ischemic infarct that leads to tissue damage in the peri-infarct regions. Studies have demonstrated that cerebral blood flow (CBF) disruption that arises from stroke can influence the lesion areas, homologous sites in the contralesional hemisphere and even in remote regions that are generally connected to the site of injury [5–8]. Thus, abnormal CBF may have an effect on the hemodynamic response [9–11], and decreased CBF in neuroanatomically intact regions may still contribute to functional deficits [12]. Previous research had suggested that different CBF alteration patterns in chronic stroke patients with different infarct locations within subcortical motor pathways, potentially provide important information for the initiation of individualized rehabilitation strategies for stroke patients involving different infarct types [13].

In this study, we sought to understand the effect of IntelliRehab (which is an intelligent medical system with customized exercises for personalized home telerehabilitation) on CBF in the whole brain and the associated region of interests (ROI). The aim of this study was therefore to measure changes in the CBF patterns of different infarction locations involving the motor pathways, and its correlation on upper limb motor function.

## Methods

We conducted a pilot, before-after single group study and measured the effects of 3-months of IntelliRehab™ use. The study was approved by Human Research Ethics Committee USM (HREC); USM/JEPeM/18030172. All subjects provided a written informed consent prior to commencing.

### Subjects

A total of eight adults (n = 8) with hemiparesis ischemic stroke were recruited from Hospital Universiti Sains Malaysia (HUSM), a suburban tertiary referral center for neurological disorders in the East Coast of Malaysia. The sample size was determined based on the mean difference (0.25) and standard deviations (0.20) from a previous study [14] calculated using PS Software [15]. In the previous study, FMA score was used as a measure to assess the participants. With a two-tailed significance level of *p* < 0.05 and power (1-β) of 0.8, the sample size needed was 8 subjects. The selection of subjects was based on purposive, convenience sampling.

**Fig. 1:**
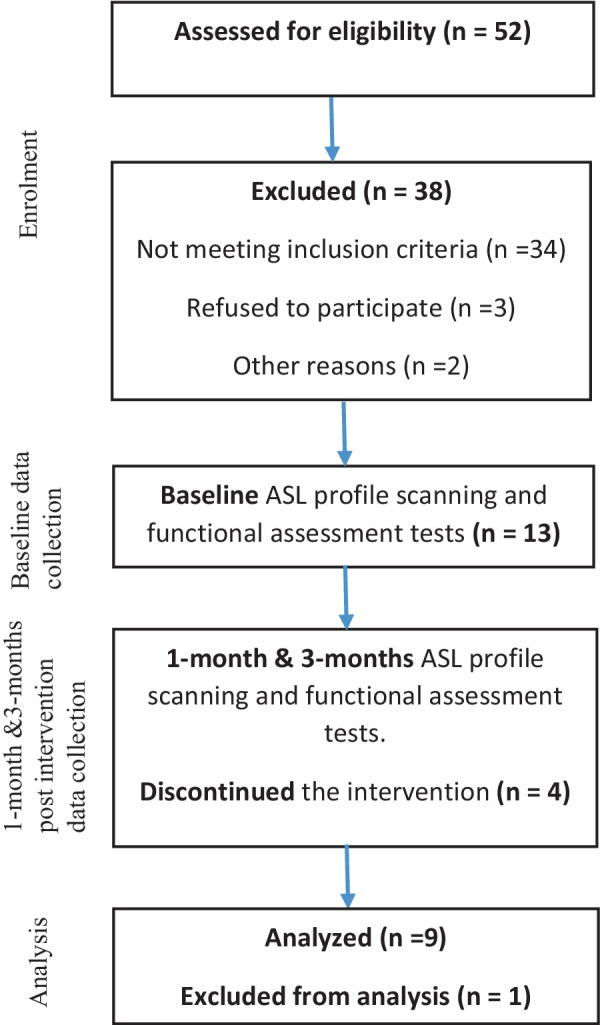
This figure demonstrates the selection of the subjects based on the inclusion and exclusion criteria for this study

Subjects recruited were hemiparetic patients who had first ischemic stroke episode within the last 4 months and were receiving conventional rehabilitation from the Rehabilitation Unit of HUSM, Kubang Kerian Kelantan at the time of recruitment. These patients were evaluated for eligibility based on the inclusion and exclusion criteria for this study as explained in Fig. 1. We included subjects who were 21-years or older, had no other known neurologic, psychiatric or developmental disabilities, demonstrated hemiparesis following an ischemic stroke, had first supratentorial unilateral ischemia in the cerebral vessels with stroke onset less four (4) months, preserved cognition, and intact vision and hearing, were medically stable, had persistent weakness involving the upper limb(s) and experience difficulty in accessing local stroke rehabilitation. We excluded patients who were unable to undergo an MRI, those with severe aphasia, apraxia, severe depression, severe shoulder subluxation, pain or shoulder dislocation, hemispatial neglect, skull breach or new stroke lesions during intervention period.

### Intervention

**Fig. 2:**
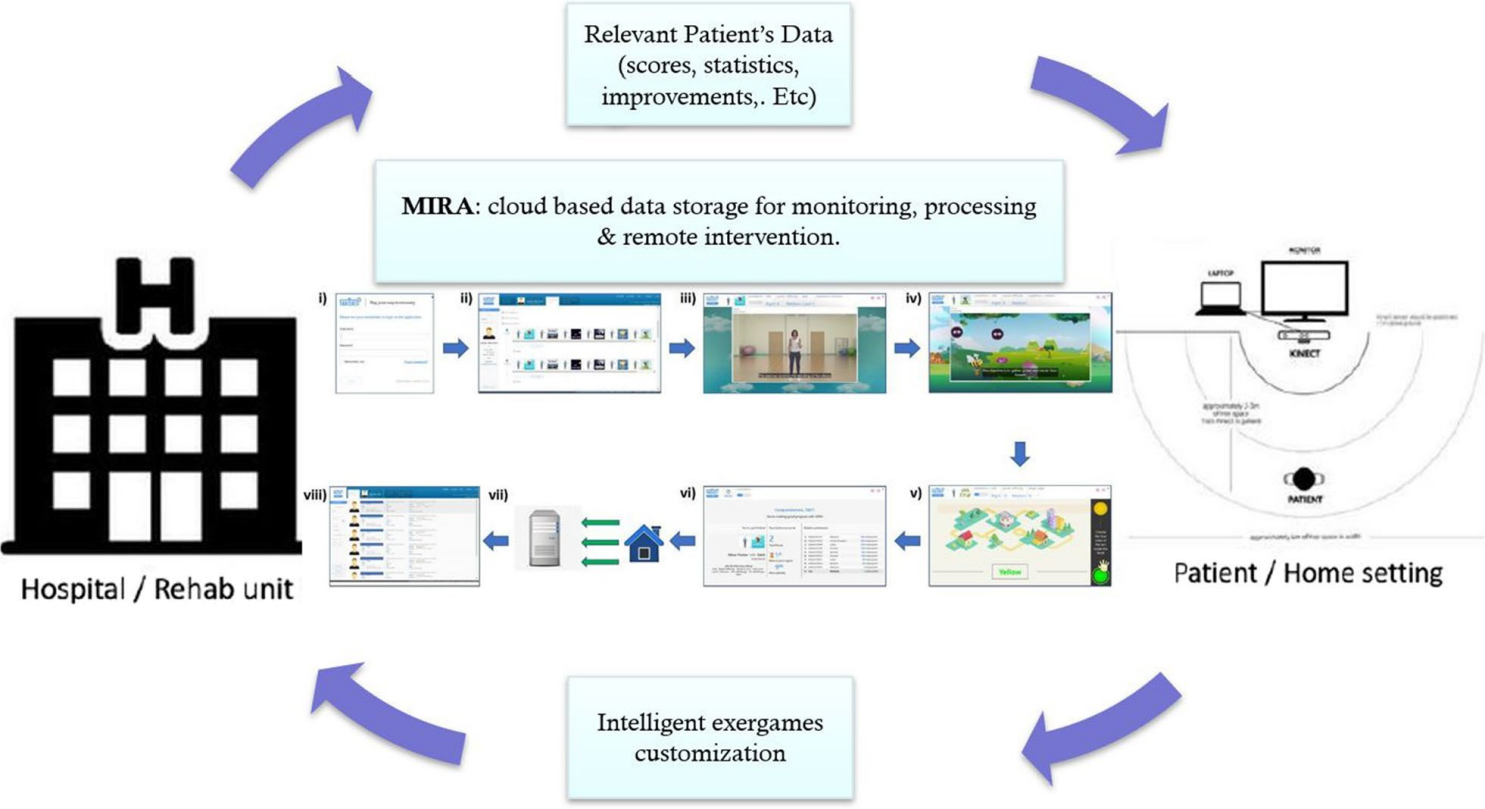
The IntelliRehab system components. IntelliRehab session consisted of; (i) Patient log-in into the MIRA platform issued with a dedicated username and password, (ii) Patient selects the arranged session (composed of 7 exergames with total duration of 21 min), (iii) Tutorial explanation of limb’s movements, (iv) Exergame’s instructions and rules description before each exergame, (v) Patient plays the exergames, (vi) Scores and ranks are given after each exergames completed, (vii) All relevant data are sent and stored in the server, viii) Data are uploaded and accessible only by the research team

IntelliRehab is augmented by customized sensor hardware that gamified physical therapy with ‘exergames’ as a mean to remotely monitor patient progress and compliance. This telerehabilitation tool consisted of (1) an intelligent virtual assistant, (2) wireless interaction sensors to capture body motions and, (3) a tool for custom exercises, all built for ease of clinical feedback. A new cloud-based platform was designed specifically to track patient data and incorporate multi-inputs, providing remote monitoring and analytics services. The IntelliRehab sessions were performed at home, where IntelliRehab tool enabled the subjects to perform their upper limb motor training at home as a telerehabilitation set-up (Fig. 2).

A cloud-based platform was designed specifically to track patient’s data and incorporate multi-inputs, providing remote monitoring and analytics services (maintained by project partner: MIRA Rehab Ltd UK). Each subject commenced first with initial training sessions (at least twice) for upper limb exercise using IntelliRehab apparatus. Monitoring and compliance were recorded with the subjects using video communication for 5 sessions every week over a period of 3-months. Each self-directed IntelliRehab session lasted between 45–60 min.

### Outcome assessments

The MRI—arterial spin labelling (ASL) profile scan and functional assessments were performed at three time points (baseline, 1-month, and 3-months). Upper limb motor function was assessed on the Fugl-Meyer Upper Extremity Assessment (FMA), and impact of stroke-specific motor changes was assessed on the Stroke Impact Scale (SIS). The FMA score is used to evaluate the levels of upper limbs motor impairment and measure the movement, coordination and reflexes of the shoulder, elbow, forearm, wrist, and hand. Thus, it represents the functional outcome of the stroke survivors during their rehabilitation course. It has previously been established and tested as a reliable, valid, and specific tests for the motor function following a stroke [16–18].

Also, SIS is a questionnaire consists of nine questions that are used to evaluate how the stroke has affected the patient’s health, quality of life and activities of daily livings (ADLs) from the patient point of view. Some of these questions emphasize on ADLs related to motor functions such as in SIS-Q1, -Q5, -Q6, -Q7, -Q8 and -Q9. SIS has previously been proved as highly reliable and valid tests to assess ADLs after stroke event [19–21].

The feasibility of the intervention including technical problems, technical support and following on self-training and training exercises was evaluated during the intervention and subjects’ responses and feedback recorded by the research team in dedicated logbooks. Questions relating to participation in the research or concerns regarding risk in relation to interventions used in the study were addressed prior to obtaining an informed consent. All ASL MRI and USM IntelliRehab Assessment Proforma data acquired was kept securely in accordance with the Data Protection Act.

### ASL MRI acquisition

MRI data acquisition was performed on an Achieva 3.0T (TX) MR system (Philips Medical System, Best, The Netherlands) using the sensitivity encoding (SENSE)-eight‐channel head coil (SENSE-Head-8) as follows. First, high-resolution sagittal T1- turbo field echo (TFE) images (that allow SENSE in slice encoding) were collected using 3D magnetization-prepared rapid acquisition with gradient echo (MPRAGE) with the following parameters: TFE shots = 88, TFE factor = 219, repetition time (TR) = 7.4 ms, echo time (TE) = 3.4 ms, Min. inversion time-delay (TI delay) = 850.8 ms, flip angle = 8°, Water-Fat Shift (WFS)/Bandwidth (BW) = 2.071/209.7 Hz/px, voxel size = 1.1 × 1.1 × 0.6 mm^3^, field-of-view (FOV) 250 × 241 × 150 mm^2^, acquisition matrix = 228 × 219, reconstruction matrix = 240, slice number = 250 slices. Secondly, 2D-pASL data were acquired using the following parameters: a single-shot 2D Fast Field Echo/Echo Planer Imaging (2D FFE/EPI) readout. SENSE was used to reduce the echo trains length by reducing the susceptibility related distortions and maximum water fat shift and short TE to keep a good signal to noise ratio (SNR). ASL Multi-Slice Single-Phase was acquired in ascending order, transverse orientation, and contained 20 slices of 6 mm-thickness with the following parameters: dynamic scans = 30, dynamic scan times = shortest, label thickness = 130 mm, label gap = 20 mm, inversion time-delay (TI delay) = 1800 ms, TR/TE = 4000 ms/20 ms, flip angle = 40°, FOV = 240 × 240 × 39 mm^2^, matrix size = 68 × 68, BW = 1260.4 Hz/px, WFS/BW = 14.943/29.1 px/Hz, Min. slice gap = 0 mm, Act. Slice gap = 0.6 mm, EPI factor = 33. A separate M0 image was not acquired; thus, the mean of the control images was used to measure M0 which was used in ASL analysis. During the resting state ASL scans, all subjects were instructed to keep their eyes closed, relaxed, and move as little as possible, without falling asleep.

### ASL processing and analysis

ASL-MRICloud is based on cloud computing using the infrastructure of MRICloud.org [22, 23], as well as computational, and storage resources on a remote server. ASL-MRICloud (https://braingps.mricloud.org/home) [22] was used to process and analyze the acquired ASL MRI data. in the following steps:

Firstly, segmentation of the acquired 3D T1 was done using T1 Multi-Atlas Segmentation option. The 3D T1 data segmentation is a hierarchical brain segmentation [24]. This type of brain segmentation can divide the brain up to 289 regions based on the age of patient and the number of atlases used during the segmentation process. T1 output was skull-stripped and presented in Montreal Neurologic Institute (MNI) template space (2 × 2 × 2 mm).

Secondly, CBF Image Calculation and Processing data were analyzed using ASL v4 option. The quantification of CBF using single‐delay ASL was analyzed through the pASL image (control and label images) series that were corrected for motion by aligning to their respective first time point [25]. This was followed by the calculation of the difference between images (control − label). Estimation of equilibrium magnetization from the control images, after accounting for all RF pulses present in the pulse sequence and by assuming a tissue T1 was used to generate a global M0 which was used in CBF quantification.

**Fig. 3:**
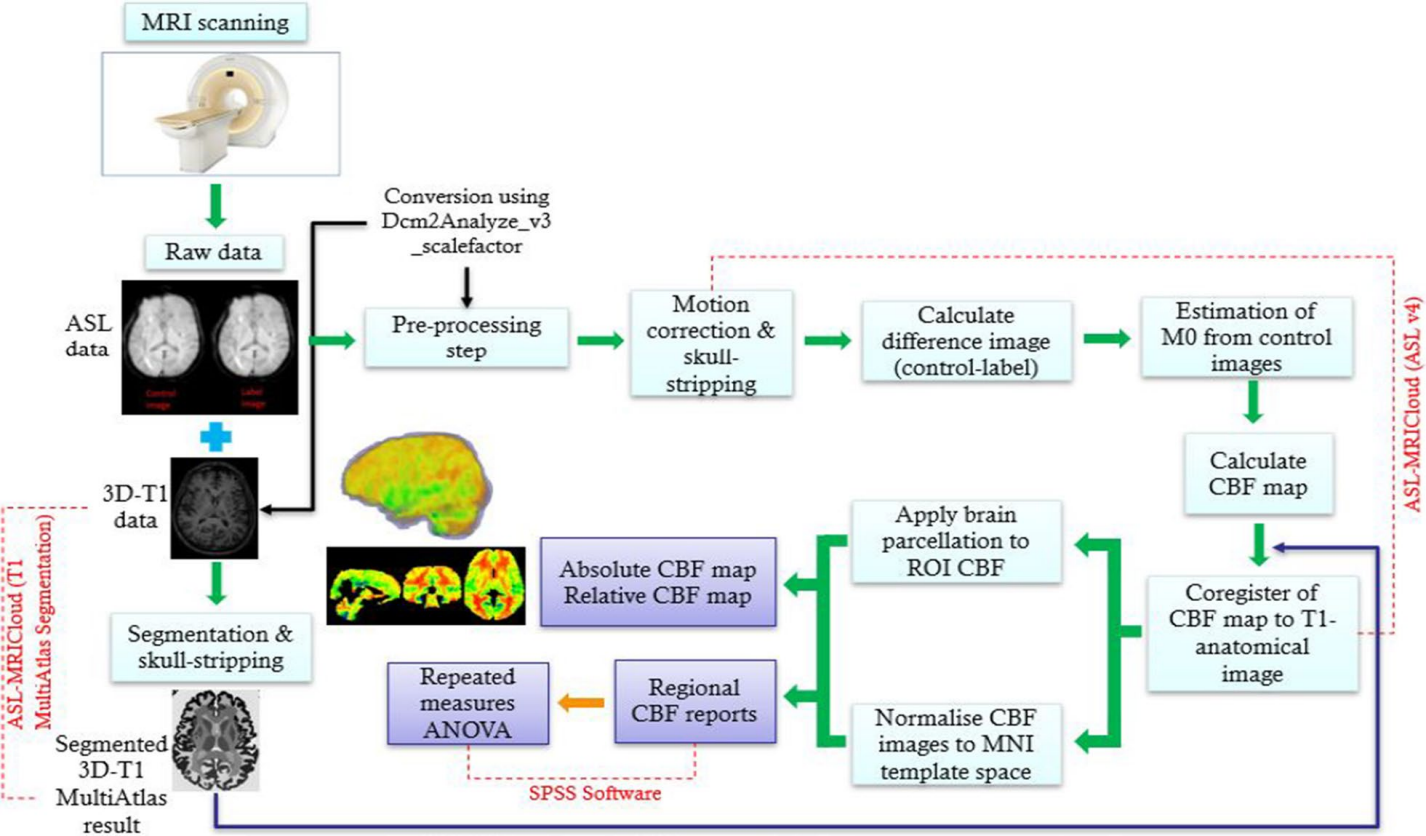
Data analysis workflow. Raw data (ASL &3D-T1) is captured using MRI scanner then converted to.img and.hdr extension. Then, a 3D-T1 data is segmented using ASL-MRICloud (T1 MultiAtlas segmentation) and ASL data is analyzed using ASL-MRICloud (ASL v4) to deduce the absolute and relative CBF map and regional CBF reports. Regional CBF is evaluated using SPSS Software (repeated measures ANOVA)

Finally, CBF maps were calculated and co-registered with T1 Multi-Atlas results to apply brain parcellation to calculate the regional CBF based on the brain segmentation. The outcome CBF results were normalized and presented in MNI template space (2 × 2 × 2 mm) with regional values of up to 289 standard brain structures. In ASL v4, these parameters were selected for the analysis depending on our current MRI protocol including; pASL in labeling scheme; 2D in acquisition scheme; acquisition timing parameters (TI1[ms] = 800, TI[ms] = 1200, slice acquisition duration [ms] = 35); option “no” in background suppression; and M0 acquisition\estimation parameters (Tissue T1 [ms] = 1165), and assumptions for CBF quantification; (Blood T1 [ms] = 1650, brain/blood partition coefficient [ml/g] = 0.9, and labeling efficiency = 0.98). The complete data workflow is shown in Fig. 3.

### Statistical analyses

Statistical Package for the Social Sciences (SPSS) version 22 (IBM Corp) was used to determine the scan time differences during three timepoints using repeated measures analysis of variance (ANOVA) test for CBF extracted values through ASL-MRICloud and for USM IntelliRehab Assessment Proforma (that represented by FMA and SIS) with a corrected threshold of *p* < 0.05. After that, the regions with significant CBF differences in the repeated measures ANOVA were extracted to be used as regions of interest (ROI). Subsequently, The Bonferroni post hoc analysis was done to determine the CBF changes and motor function changes represented by FMA and SIS during the 3-months duration (at three time points (baseline, 1-month, and 3-months) of IntelliRehab. Bonferroni correction compensates for increase of type 1 error that resulted from the likelihood of incorrectly rejecting a null hypothesis by testing each individual hypothesis at a significance level of 0.05/m where m is the number of hypotheses [26, 27]. The *p value* in the Bonferroni correction for ROI, FMA and SIS has been corrected using SPSS software*.* Finally, Pearson correlation test was used to determine the relationships between the significant afflicted brain regions of ASL Profile and the significant FMA and SIS tests. All *p* values were 2-tailed. Values of *p* < 0.05 were considered statistically significant.

## Results

### Demographic and clinical data

**Fig. 4:**
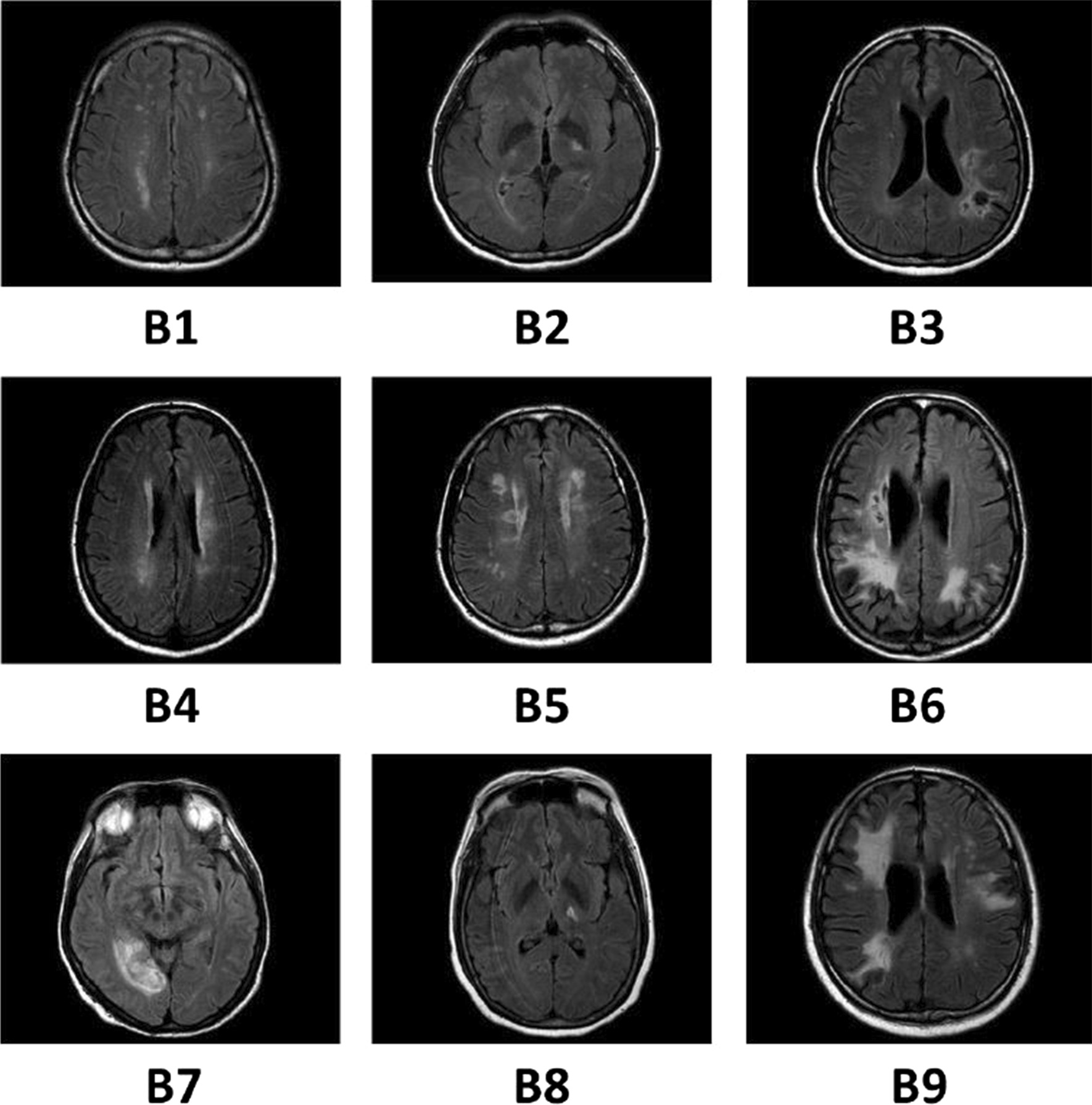
Individual FLAIR images among stroke patients in IntelliRehab in the axial view. The panel **B1** to **B9** shows the slice with maximum infarct volume/hyperintensities. Each subject was coded by a serial number

Subjects ages were between 38 to 72 years (mean: 55.8 ± 7.9) and were mostly male (n = 7; 87.5%). The detailed demographic and clinical data of our recruited subjects are shown in Table 1. All the subjects were right hand dominant which have been tested by a reliable, valid test called Edinburgh handedness inventory test [28, 29]. Also, the axial view of each individual FLAIR images among stroke patients have been demonstrated in (Fig. 4).

**Table 1:** Demographic and clinical data summary of the subjects

Gender	Male	Female	
	77.8%	22.2%	
Side of hemiparesis	Right	Left	
	66.7%	33.3%	
Duration from stroke onset	Mean	1 month	2 months
	1.3 month	66.7%	33.3%
Risk factors	HTN	HCL	Smoker
	88.9%	66.7%	55.6%
	DM	Coagulopathy	
	33.3%	11.1%	
Stroke lesion location	CR	PLIC	OR
	33.3%	22.2%	22.2%
	PR		
	22.2%		
Stroke type	Subcortical	Cortico-cortical	Cortical
	62.5%	25%	12.5%
Handedness*	Right	Left	
	100%	0%	

*As determined by Edinburgh Handedness Inventory. *HTN* Hypertension, *DM* Diabetes Mellitus, *HCL* Hypercholesterolemia, *CR* Corona radiata, *PR* Parietal region, *PLIC* Posterior limb of internal capsule, *OR* Occipital region

### Whole brain analyses of CBF changes in the intellirehab using ASL-MRIcloud

Repeated measure ANOVA evaluating overall CBF changes following IntelliRehab use (across the three timepoints) showed no significant CBF differences in ipsilesional or contralesional ischemic stroke group. However, three afflicted brain regions showed significant CBF differences between timepoints, these were two cortical regions (left middle fronto-orbital gyrus (MFOG_L) and right inferior temporal gyrus (ITG_R)), and one subcortical region i.e. left superior temporal white matter (STWM_L) with *p* < 0.05*.* Among the whole brain analyses in chronic stroke survivors under IntelliRehab; we found MFOG_L part of the contralesional cerebral cortex (*p* = 0.049); ITG_R part of the ipsilesional cerebral cortex (*p* = 0.008) and STWM_L part of the contralesional White matter (*p* = 0.026) had significant CBF alterations.

### Voxel-wise region of interest analysis

**Fig. 5:**
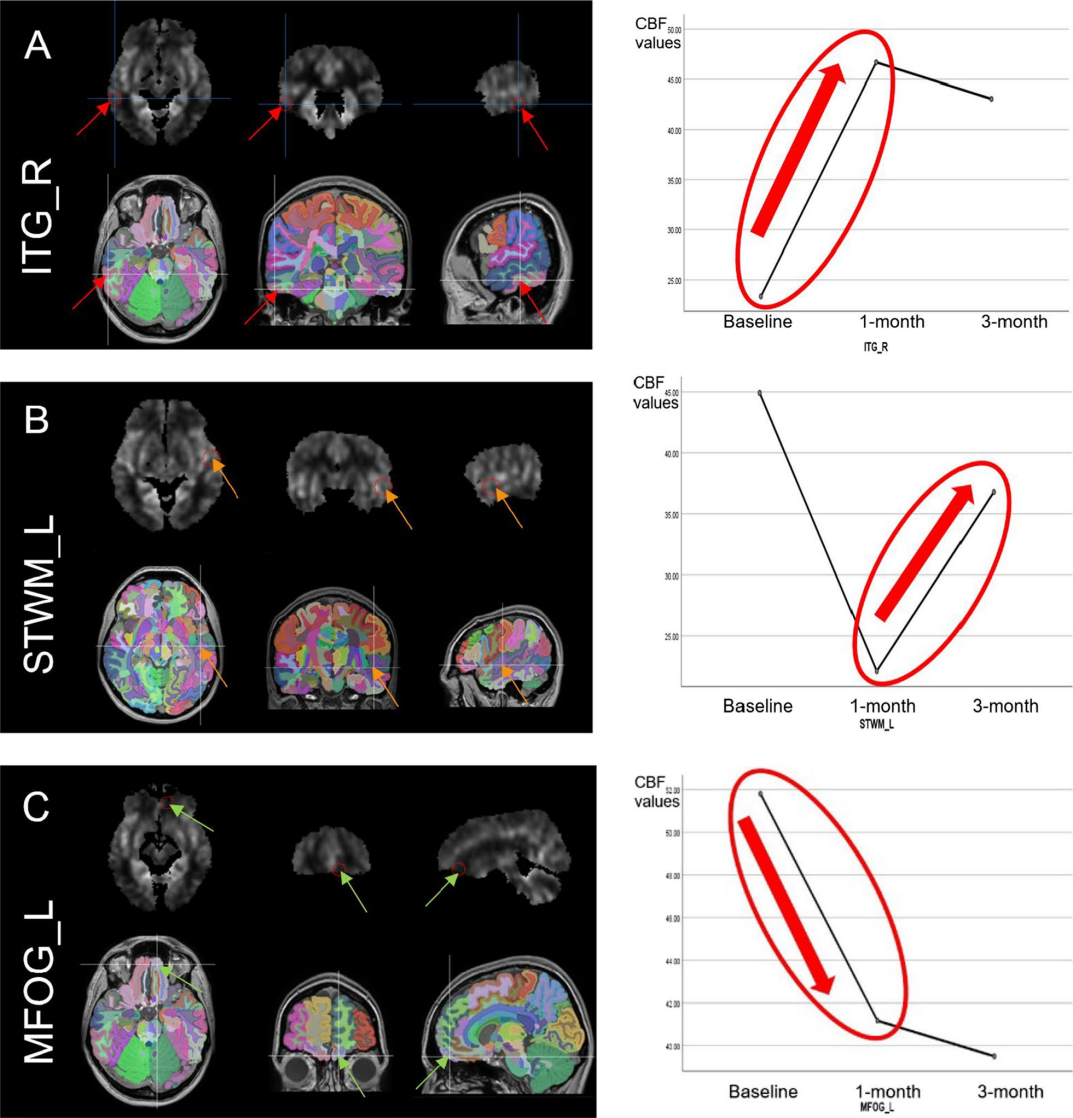
Regions of interest (ROI) that showed significant CBF differences and their representation on Profile plotting graph. **A** shows a significant rise in regional CBF of right inferior temporal gyrus (ITG_R) after 1-month and **B** indicates the increment of Regional CBF in left superior temporal white matter (STWM_L) after 3-months, while **C** reveals significant reduction of regional CBF in left middle fronto-orbital gyrus (MFOG_L) after 1-month rehabilitation

The brain regions that displayed significant CBF differences were defined as ROI for further investigation via post hoc using Bonferroni test as explained in the method section. We found that after the 1-month of rehabilitation, subjects had significant increment of the regional CBF in ITG_R relative to the baseline (*p* = 0.044). Subjects also showed a trend toward increased in the regional CBF (*p* = 0.01) in the STWM_L after 3-months of rehabilitation relative to 1-month. In contrast, IntelliRehab subjects showed significantly decreased in the regional CBF in the MFOG_L after 1-month of rehabilitation (*p* = 0.012) compared to the baseline (Fig. 5).

Among the identified significant brain regions of CBF alteration in IntelliRehab group, two regions of the cerebral cortex have different effect on regional CBF after 1-month of rehabilitation. MFOG_L which lies in the ipsilesional frontal cortex, had significant decline of regional CBF after 1-month of rehabilitation (mean = 51.8 ± 10.2; *p* = 0.012 < 0.05) compared to the baseline (mean = 41.2 ± 9.6). However, ITG_R which presents in the contralesional temporal cortex had significant rise of regional CBF after 1-month of rehabilitation (mean = 46.7 ± 18.5; *p* = 0.044 < 0.05) compared to the baseline (mean = 23.3 ± 8.2).

Furthermore, we noticed that STWM_L brain region which lies in the ipsilesional temporal white matter had significant rise of regional CBF after 3-months of rehabilitation (mean = 36.8 ± 9.8; p = 0.001 < 0.05) compared to the 1-month (mean = 22.1 ± 9.6). This interesting effect of IntelliRehab on the regional CBF may be due to the integration of ITG_R and STWM_L regions during IntelliRehab sessions more than MFOG_L or due to functional reorganization, and increased connectivity in ITG_R and STWM_L regions.

### Effect of intellirehab on the motor function

FMA of IntelliRehab subjects showed a significant increment after 3-months of rehabilitation (mean = 62.4 ± 3.8; *p* = 0.048 < 0.05) compared to the 1-month (mean = 57.9 ± 7.4). This result reflects the effectiveness of IntelliRehab system in improving the functional outcome after 3-months which led to an increase in the ADLs of stroke subjects.

**Fig. 6:**
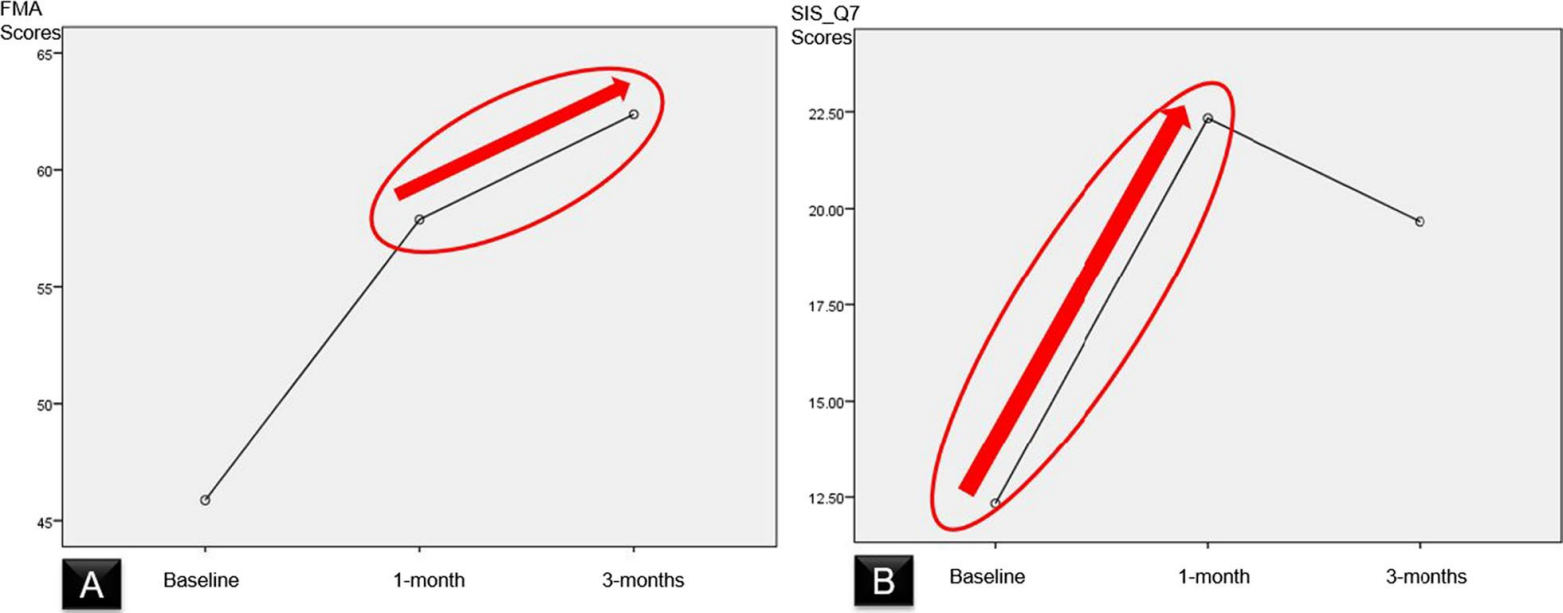
Profile plotting graphs for Fugl-Meyer Upper Extremity Assessment (FMA) and Stroke Impact Scale (SIS) respectively. **A** indicates the increment of FMA from baseline to 3-months, whereas **B** shows the increment in SIS_Q7 after 1-month rehabilitation

Intra-group analysis showed significant increase in FMA (*p* = 0.014) and SIS-Q7 (*p* = 0.025) scores. There was a significant increment of FMA score (*p* = 0.048) after 3-months of IntelliRehab compared to the 1-month. Furthermore, SIS_Q7 score displayed a significant rise after 1-month of IntelliRehab (*p* = 0.000) relative to the baseline (Fig. 6).

### Correlation analyses between CBF changes and motor function

**Fig. 7:**
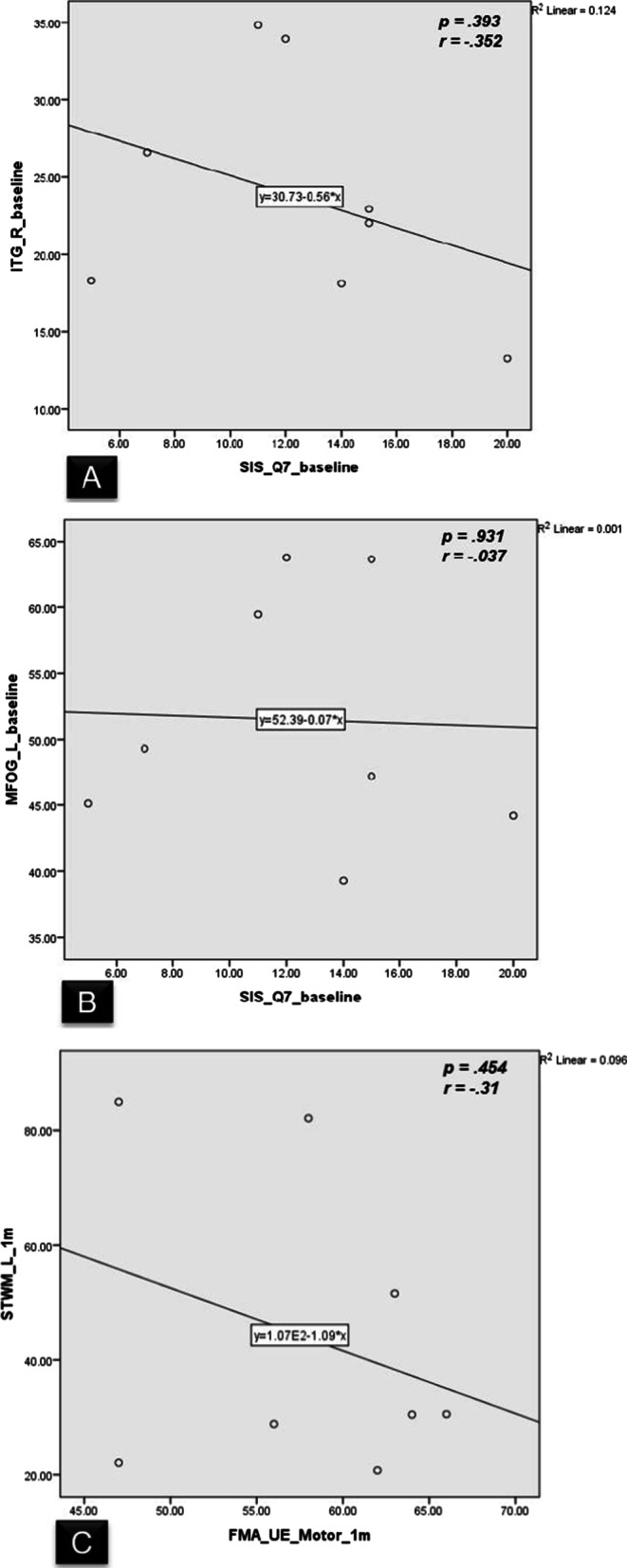
Correlations between the CBF changes and motor functional scores during IntelliRehab. **A**–**C** show no significant correlation between 3 ROI CBF with motor function

Pearson correlation analyses were used to investigate the relationships between the CBF changes and motor function (FMA and SIS-Q7 scores) for stroke survivors who were under IntelliRehab in brain regions showing significant CBF changes and significant motor function scores during 3 sets of assessments. We did not find any correlation between either the rise of regional CBF of ITG_R (*p* = 0.393) or the drop of regional CBF of MFOG_L (*p* = 0.931) brain regions with the significant rise of SIS-Q7 after 1-month of rehabilitation. Also, no significant correlation between regional CBF rise in STWM_L and FMA (*p* = 0.454) under IntelliRehab during (1-month – 3-months duration of rehabilitation) (Fig. 7).

## Discussion

In this study, the IntelliRehab system for upper extremity rehabilitation seemed to influence CBF as shown by the intra-group regional CBF changes in two cortical regions and one white matter region within our 3-months study duration. Furthermore, the motor function represented by FMA and SIS exhibited significant improvement to suggest the potential benefit of IntelliRehab as a tool of home-based telerehabilitation.

The human brain is a complex organ that consists of structural connections and intensive functional interactions between cortex and subcortical structures. Such an architecture enables our brain to coordinate complex and delicate sensory-motor and autonomic functions and poses plasticity to recover from stroke. However, it is still unclear how cortical and subcortical structures and functions change in the presence infarctions in motor pathway of the brain. It has been noted that motor impairment, involving upper limb hemiparesis, is the most common deficit in up to 80% of stroke patients [30, 31]. That impairment may arise from damage to the subcortical structures (e.g., basal ganglia), or to cortical regions (e.g., primary motor cortex), and/or the corticospinal tract [32]. This study has sought to better understand these relationships, between changes in the CBF patterns of different infarction locations involving the motor pathways, as well as measure any potential effect such changes may have on upper limb motor function.

In addition, numerous prospective clinical studies indicate that subcortical strokes primarily affect structures such as basal ganglia, white matter, and/or the thalamus [33–35]. Moreover, these studies also reported that pure cortical strokes accounted for about 15% of all strokes while subcortical-only and combined cortico-subcortical strokes occurred at a much greater frequency. Few studies have directly compared the motor outcome recovery following cortical versus subcortical strokes. Some studies exposed that the cortical stroke survivors showed better motor recovery than those with a purely subcortical stroke (e.g., basal ganglia, internal capsule) [36, 37]. Nonetheless, others have reported no apparent effect of lesion location (cortical/subcortical) on the functional outcome [38, 39].

In the intra-group comparison for CBF changes for both ipsilesional and contralesional analogy, we did not find any statistically significant results. However, we found that in the contralesional cerebrum, the CBF mean for IntelliRehab showed increment after 3-months. In addition, the ipsilesional cerebral hemisphere revealed a decrease of CBF mean after 1-month then increase after 3-months. These findings of no significant intra-group differences on the ipsi- or contralesional CBF of the cerebral hemisphere might be due to the varying lesion locations between the stroke survivors and some of them had more than one lesion (47%). Brumm’s research team investigated the CBF alterations in three cortical chronic stroke survivors with right-sided hemiparesis individually compared to younger and elderly participants[12]. They noted that there was a hypoperfusion in areas throughout the left cerebral hemisphere of each stroke survivor, and especially in peri-lesional regions. Results for those three stroke survivors in their study indicated significantly greater right hemisphere CBF than left hemisphere CBF and significantly greater CBF within the right hemisphere peri-lesional homologue area as compared to the left hemisphere peri-lesional area. Moreover, CBF ratios of the entire left hemisphere versus right hemisphere demonstrated lower CBF within the intact portions of the insulted left hemisphere as compared to the intact right hemisphere [12].

Ischemic infarcts may influence the blood supply in the regions affected by the infarct lesion, and thus causing brain tissue damage in the that region and, in the peri-infarct regions. Several studies have found that stroke lesions cause CBF disruption at the lesion areas, the homologous sites in the contralesional hemisphere and even in remote regions that are generally connected to the site of injury [5, 6, 40]. Previous research has shown that different infarct locations can cause different patterns of secondary brain injury and functional reorganization that resulted from lesion localization which in turn, influenced the functional outcome [4]. Therefore, cerebral cortical impairment directly leads to damage the cortical functional area, but subcortical damage may influence the cortical-subcortical circuit, resulting in secondary cortical function damage and reorganization [8, 41].

Part of the explanations to the cortical damage and reorganization is due to metabolic abnormalities which are recognized to influence functional outcome and prognosis after ischemic stroke. In this context, CBF plays an important role in mirroring cerebral metabolic demand and neuronal function [42]. Thus, abnormal CBF reflects the underlying hemodynamic response [9–11], and decreased CBF in neuroanatomically intact regions may precipitate another functional deficits [12]. In this study we explored the changes of CBF of chronic stroke subjects using IntelliRehab and their impacts on the motor function over 3-months duration.

Previous study found that basal ganglia stroke patient displayed significantly increased CBF in the contralesional putamen relative to normal controls and significantly decreased CBF in the ipsilesional sensorimotor cortex [43], ipsilesional thalamus and contralesional cerebellum, which may be ascribed to an influence of the ischemic lesion directly on the blood supply of peri-infarct regions[13]. Also, there is a structural impairment in the ipsilesional sensorimotor cortex of basal ganglia stroke patients and in the cerebellum of pontine stroke survivors [44]. Pappata and colleagues reported in their positron emission tomography (PET) scan study that a significant ipsilesional cortical and contralesional cerebellar hypometabolism in concurrence with demonstrated atrophic alteration in these regions, which were attributed to retrograde and anterograde degeneration [45–47]. These results are supportive of the findings in this study where IntelliRehab system influenced the intra-group regional CBF alterations within 3-months duration of rehabilitation in the mentioned three regions of interest. Moreover, Wiest and colleagues had found that a poor recovery of motor function in the chronic stage of stroke patients due to a sustained CBF decline in the ipsilesional sensorimotor cortex [43]. This suggests implementation of different rehabilitation strategies with different infarct types at the earliest stage of stroke may influence CBF progress [13].

Furthermore, we noticed that STWM_L brain region which lies in the ipsilesional temporal white matter had significant rise of regional CBF after 3-months of rehabilitation. This interesting effect of IntelliRehab on the regional CBF may be due to the integration of ITG_R and STWM_L regions during IntelliRehab sessions more than MFOG_L or due to functional reorganization, and increased connectivity in ITG_R and STWM_L regions. Thus, our findings suggest a potential functional compensation of ITG_R and STWM_L for the impaired brain regions[13]. Also, this might suggest that the neuroanatomically structurally intact MFOG_L brain region had a connection with the infarction site which could lead to decrease CBF in that afflicted brain region. This is in agreement with previous studies that found that stroke lesions cause CBF disruption at the lesion areas, the homologous sites in the contralesional hemisphere and even in remote regions that are generally connected to the site of injury [5, 6, 40]. Decreased CBF in neuroanatomically intact regions may precipitate another functional deficit [12], and thus, regions in the ipsilesional hemisphere would exhibit a greater decline in the regional CBF compared to the contralesional hemisphere. can suggest the link between affected and unaffected regions as determined from. In addition, any subject who had severe dysphasia was excluded from our study and those who had mild to moderate dysphasia were recruited in the study and may have received speech therapy as part of the overall stroke rehabilitation program for the individual patient. A study done by Hillis et al. (2001c) during acute strokes showed a strong correlation between hypoperfusion in the posterior superior temporal gyrus and impaired word comprehension abilities[48]. This may suggest an effect of IntelliRehab on these three ROIs in the temporal lobes.

Correspondingly, these results are in line with previous studies that showed functional damage or hypoperfusion mainly in the motor system in chronic subcortical stroke patients and the decreased CBF contributes to their motor and cognitive functional impairment [12, 13]. Our results suggest that CBF changes in ipsilesional or contralesional hemisphere may highlight remote neuronal plasticity and functional reorganization. This trend may also be attributed to a relative increase in physical activity in daily life, resulting in an increase activation of functional areas to compensate for the damaged ipsilesional area [49–51].

Virtual reality-based rehabilitation (VRBR) has been used in stroke rehabilitation for over the last 10 years [52] to enable simulated practice of functional tasks at a higher doses than traditional therapies [53, 54]. More recently, these virtual reality interventions built with individual customization and commercial gaming systems have demonstrated significant gains in upper limb function, ADLs performance, and balance, as compared with other interventions, which suggests a positive impact with their relevance for training of community ambulation [55]. Kinect-based virtual reality system which can be regarded as a part of VRBR, also can potentially boost the home-based rehabilitation through usability, gamification, automated feedback features, and cost-effectiveness as compared with complex robotic systems. IntelliRehab is an example of Kinect-based virtual reality system that was integrated into customized exergames platform by MIRA UK (our research collaborator) to improve the delivery of home-based rehabilitation.

The primary motor function was assessed using FMA, which showed a significant improvement following receiving rehabilitation using IntelliRehab. Our research results are consistent with the previous studies that demonstrated the moderate advantages of VRBR on body functions of the upper limb [56, 57] and lower limb [58], and on activities when compared to standard rehabilitation in stroke patients. Randomized control trials (RCTs) found that VR was more effective than conventional therapy, as well as significantly more effective than no therapy in improving upper limb function [59]. VRBR has also been shown to improve ADL function when compared to more conventional therapy approaches [60–66] which supports our current results related to the use of IntelliRehab system. FMA of IntelliRehab subjects showed a significant increment after 3-months of rehabilitation compared to the 1-month. This result reflects the effectiveness of IntelliRehab system in improving the functional outcome after 3-months which led to an increase in the ADLs of stroke subjects.

On the other hand, the secondary motor function was assessed using SIS score for stroke patient under IntelliRehab had a significant difference in Question-7 (SIS_Q7) that exhibited a significant increment after 1-month of rehabilitation compared to the baseline. SIS-Q7 signifies the patient’s ability to use his/her hand that was most affected by the stroke. Our results in agreement with previous study findings that showed similar significance in SIS-Q7 after one-month in their stroke subjects [67].

Our study sought to test the correlation between CBF changes in the ROI and motor function on FMA and SIS, following subject participation in using the IntelliRehab system. While we found no significant correlation, one study reported a significant positive correlation between their subjects with basal ganglia stroke that displayed a decreased CBF of the ipsilesional sensorimotor cortex whilst subjects with pontine stroke displaying a decreased CBF of the ipsilesional cerebellum[13]. In their study, they had compared the chronic subcortical stroke survivors with healthy controls then correlated them with FMA scores. In our research we had different lesions sites (cortical, subcortical, and cortico-subcortical infarcts) who were followed for three months then compared between timepoints (baseline, 1-month, and 3-months) and related with FMA and SIS scores.

## Conclusion

In conclusion, we found that using the IntelliRehab system for upper extremity rehabilitation influenced cerebral blood flow changes in two cortical regions and one white matter region over our 3-months study duration. Furthermore, the motor function was found to improve in the subjects, as measured using the FMA, which suggests potential benefit of using IntelliRehab as a tool of home-based telerehabilitation. While there were no significant correlations between the CBF changes and motor function within our study, this may have been influenced by the chronic nature of the recruited stroke subjects. Further research investigating the relationships between cerebral hemispheric changes, motor function, and intensive rehabilitation is warranted.

## Data Availability

The data sets used and/or analyzed during the study are available from the first author on reasonable request**.**

## Abbreviations

CBF: Cerebral blood flow
2D-pASL: Two-dimensional pulsed Arterial Spin Labelling
ROI: Regions of interest
FMA: Fugl-Meyer Upper Extremity Assessment
SIS: Stroke Impact Scale
ITG_R: Right inferior temporal gyrus
STWM_L: Left superior temporal white matter
MFOG_L: Left middle fronto-orbital gyrus
CVD: Cerebrovascular disease
DLAYs: Disability-adjusted life-years
HUSM: Hospital Universiti Sains Malaysia
ADLs: Activities of daily livings
TFE: Turbo field echo
MPRAGE: Magnetization-prepared rapid acquisition with gradient echo
TR: Repetition time
TE: Echo time
T1 delay: Inversion time-delay
WFS: Water-Fat Shift
BW: Bandwidth
2D FFE/EPI: Single-shot 2D Fast Field Echo/Echo Planer Imaging: SNR: signal to noise ratio
ANOVA: Repeated measures analysis of variance
VRBR: Virtual reality-based rehabilitation
RCTs: Randomized control trials
SD: Standard deviation
